# Paradoxical psoriasiform dermatitis involving the nasal mucosa in the setting of infliximab for inflammatory bowel disease

**DOI:** 10.1016/j.jdcr.2024.07.023

**Published:** 2024-08-27

**Authors:** Subin Jacob-George, Kaitlyn Yim, April Deng, F.N.U. Nutan, Nikki A. Levin

**Affiliations:** a Department of Dermatology, UMass Memorial Medical Center, Worcester, Massachusetts; b Division of Anatomic Pathology, University of Massachusetts Chan Medical School, Worcester, Massachusetts

**Keywords:** anti-TNFs, IBD, inflammatory bowel disease, paradoxical adverse events, paradoxical psoriasis, psoriasiform dermatitis, psoriasis, tumor necrosis factor alpha antagonists

## Introduction

Tumor necrosis factor alpha antagonists (anti-TNFαs) have been widely used to treat autoimmune conditions including psoriasis, psoriatic arthritis, ankylosing spondyloarthropathies, rheumatoid arthritis, and inflammatory bowel disease. Although anti-TNFαs are more targeted in their actions compared to traditional immunosuppressive medications, they have several adverse effects. These include injection site reactions, infusion reactions, and increased risk of infection. Less commonly reported adverse effects include demyelinating disease, congestive heart failure, malignancies, induction of autoimmunity, and cutaneous adverse reactions, including paradoxical psoriasis. This phenomenon includes worsening of pre-existing psoriasis or onset of new psoriasiform eruption in a patient with no prior history of psoriasis or psoriatic arthritis in the setting of anti-TNF use. The postulated pathogenesis involves unopposed production of interferon-alpha by cutaneous plasmacytoid dendritic cells due to the changes in cytokine balance. Paradoxical psoriasis has occurred in approximately 5% of patients on chronic TNF antagonist treatment.^1^

There are 57 cases from the French Pharmacovigilance Database and 184 cases from other literature showing psoriasiform dermatitis induced by anti-TNFαs (infliximab, etanercept, adalimumab). The psoriasiform eruptions reported were palmoplantar pustular psoriasis (33.3% in French Pharmacovigilance Database, 42.9% in other literature), plaque psoriasis (15.8%, 14.7%), generalized pustular psoriasis (5.3%, 7.6%), inverse psoriasis (1.7%, 1.1%), and scalp psoriasis (7.0% French Pharmacovigilance Database, none in other literature).^2^ Of the 3 anti-TNFαs, about 50% of the cases were due to infliximab. Notably, women aged 40-50 years old were most often affected. There was a higher incidence of de novo psoriasiform dermatitis versus an exacerbation of pre-existing psoriasis.^2^

Paradoxical psoriasiform dermatitis has been observed to occur on the scalp, flexural, and extensor surfaces of extremities and trunk, skin folds, and on the palms of hands and soles of feet.^3^ Nails are affected less frequently with typical clinical findings of onycholysis, discoloration, and nail pitting. Mucocutaneous involvement has not been observed as a result of paradoxical psoriasis. In the majority of cases, the psoriasiform dermatitis resolved with discontinuation of the anti-TNFαs, changing treatment to a non-TNF inhibitor biologic therapy, or symptomatic treatment with topical corticosteroids. Resolution of the psoriasiform dermatitis lesions can take weeks to months depending on the severity and the efficacy of alternative treatments. We report an unusual presentation of TNF-alpha inhibitor-associated psoriasiform dermatitis presenting initially in the nares, specifically the nasal vestibule and mucosa.

## Case report

**Fig 1:**
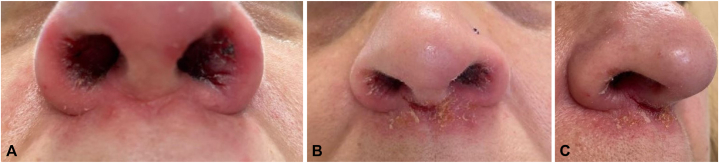
Erythematous, scaly rash with deep fissures and crusting of the nasal vestibule, upper lip. Image shows persistent rash despite 6+ months of treatment for suspected impetigo with mid-high potency topical corticosteroids, antifungal creams, oral antibiotics, or oral antiviral medications.

**Fig 2:**
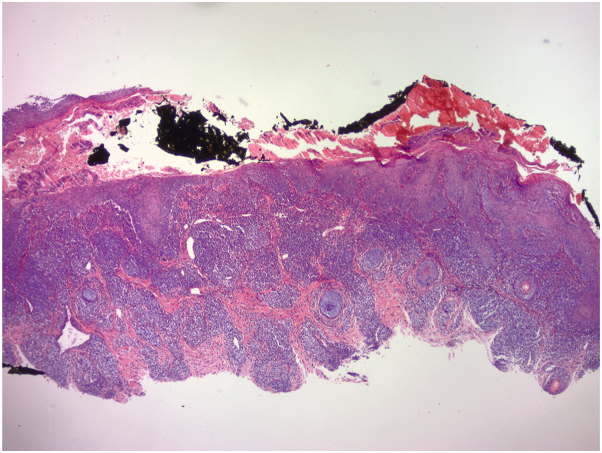
The specimen shows a psoriasiform dermatitis with subcorneal pustules, impetiginized neutrophilic crust, and a dense perivascular and interstitial lymphohistiocytic infiltrate with neutrophils and eosinophils. Gram stain shows gram-positive cocci in the crust. The differential diagnosis includes a psoriasiform drug eruption or impetigo.

A 50-year-old woman with inflammatory bowel disease and no prior personal or family history of psoriasis presented with a 1-year history of an erythematous, scaly rash with deep fissures and crusting of the nasal vestibule, ears, and scalp (Fig 1). She had been on infliximab for ulcerative colitis for over 10 years. There was no history of preceding viral illness or streptococcal infection. Incisional biopsy of the left nasal vestibular mucosa performed by the patient’s otolaryngologist revealed squamous epithelium with acanthosis, parakeratosis with scale crust, and dense lymphoplasmacytic infiltrate in the subepithelial stroma (Fig 2). The case was reviewed by dermatopathology, confirming psoriasiform dermatitis with subcorneal pustules and Gram-positive cocci in the scale crust, reflecting possible concomitant impetigo.

**Fig 3:**
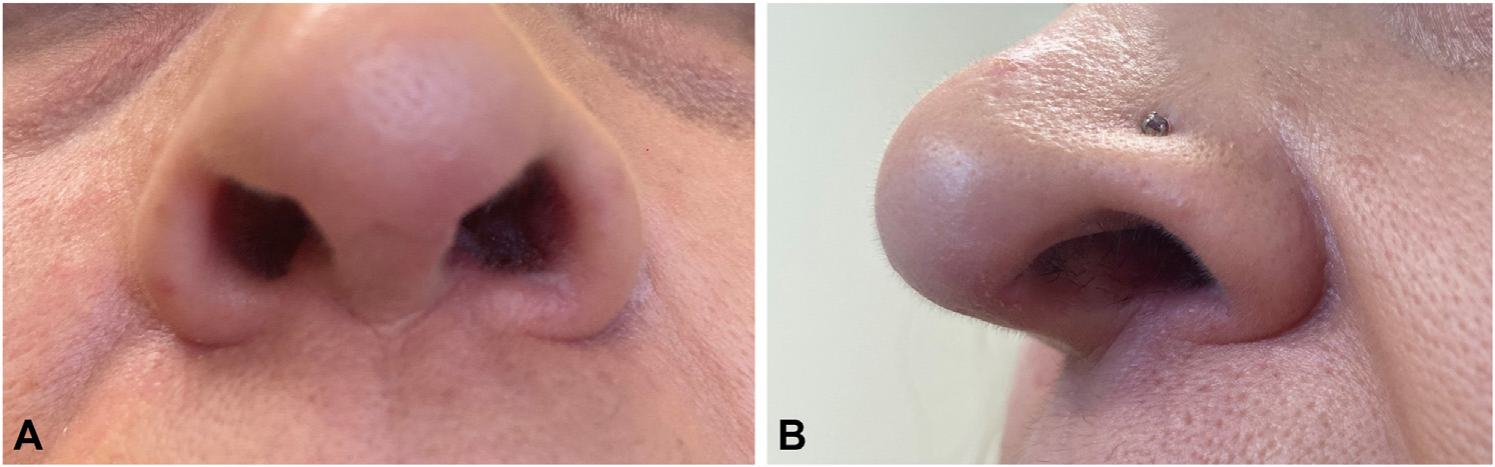
Complete resolution of nasal mucosal rash 16 weeks s/p dose 2 of ustekinumab.

Based on the clinical presentation, the patient had been treated for suspected impetigo for close to 1 year with alternating rounds of topical and oral antibiotics, high-potency topical corticosteroids, antifungal creams, and oral antiviral medications with minimal to no improvement. Based on the biopsy results, her gastroenterologist discontinued infliximab and started ustekinumab. Six weeks after stopping infliximab, she had significant improvement of the psoriasiform dermatitis, fissuring, and crusting. She still had a mildly erythematous and scaly rash on the upper lip and a similar rash behind the right ear with mild fissuring. She also had new scaly, hyperkeratotic erythematous plaques on the vertex scalp which were treated with over-the-counter salicylic acid shampoo daily and fluocinolone 0.01% external oil. After her second dose of ustekinumab, she had complete resolution of the nasal mucosal lesions while maintaining good control of ulcerative colitis (Fig 3). Subsequently, she discontinued ustekinumab due to lack of efficacy and started risankizumab and methotrexate with better control.

## Discussion

We present an unusual case in which paradoxical psoriasiform dermatitis affected the nasal mucosa. This contrasts with the known palmoplantar, plaque, and guttate psoriasiform lesions typically seen in paradoxical psoriasis. Mucosal psoriasiform dermatitis is extremely rare with a total of 64 cases affecting the mucosa observed in literature.^4^ The reports in the review identified psoriasiform lesions occurring on the lips, buccal mucosa, gingivae, palate, tongue, and floor of the mouth.^5^ Very rare cases of circinate psoriasis involving the genitalia have also been reported.

There are rare reports of several cutaneous diseases with primarily mucosal involvement. Mucosal involvement has been seen with mycosis fungoides, with a total of 36 cases, most of which involved the oral, rather than nasal, mucosa.^6^ Granulomatosis with polyangiitis has been known to commonly cause sinonasal symptoms, occurring in 85% patients and 25% of patients who only have sinonasal involvement starting in the nasal septum and then spreading to the paranasal sinuses.^7^ Case review of 2319 patients with sarcoidosis showed biopsy-proven nasal mucosal involvement in 1% of patients, which presented with friable nasal mucosa, nasal polyps, or submucosal nodularity.^8^

An observational and cross-sectional study involving 73 systemic lupus erythematosus patients with stable disease activity showed the most common nasal symptoms were congestion (31.5%), nasal itchiness (26.0%), runny nose (20.5%), and nasal dryness (19.2%). Almost half (42.9%) of the subjects had nasal mucosal abnormalities.^9^ Nasal involvement (75%) is commonly seen in Churg-Strauss syndrome, with clinical findings manifesting as allergic rhinitis and chronic rhinosinusitis with or without polyps.^10^ Given our patient’s clinical presentation, histological findings confirming psoriasiform dermatitis, and improvement after discontinuing anti-TNF treatment, we feel that her nasal mucosal lesions are most consistent with paradoxical psoriasis. This case demonstrates that paradoxical psoriasiform dermatitis associated with anti-TNF use may involve the nasal mucosa and is a reminder to broaden the differential diagnosis when considering nasal mucosal lesions.

## Conflicts of interest

None disclosed.
